# Prediction of plaque progression using different machine learning models of pericoronary adipose tissue radiomics based on coronary computed tomography angiography

**DOI:** 10.1016/j.ejro.2025.100638

**Published:** 2025-02-15

**Authors:** Jingjing Pan, Qianyu Huang, Jiangming Zhu, Wencai Huang, Qian Wu, Tingting Fu, Shuhui Peng, Jiani Zou

**Affiliations:** a Medical College of Wuhan University of Science and Technology, Wuhan, Hubei 430065, China; b Department of Radiology, General Hospital of Central Theater Command of People's Liberation Army, Wuhan, Hubei 430070, China

**Keywords:** Plaque progression, Pericoronary adipose tissue, Radiomics, Machine learning, Coronary computed tomography angiography

## Abstract

**Objectives:**

To develop and validate the value of different machine learning models of pericoronary adipose tissue (PCAT) radiomics based on coronary computed tomography angiography (CCTA) for predicting coronary plaque progression (PP).

**Methods:**

This retrospective study evaluated 97 consecutive patients (with 127 plaques: 40 progressive and 87 nonprogressive) who underwent serial CCTA examinations. We analyzed conventional parameters and PCAT radiomics features. PCAT radiomics models were constructed using logistic regression (LR), K-nearest neighbors (KNN), and random forest (RF). Logistic regression analysis was applied to identify variables for developing conventional parameter models. Model performances were assessed by metrics including area under the curve (AUC), accuracy, sensitivity, and specificity.

**Results:**

At baseline CCTA, 93 radiomics features were extracted from CCTA images. After dimensionality reduction and feature selection, two radiomics features were deemed valuable. Among radiomics models, we selected the RF as the optimal model in the training and validation sets (AUC = 0.971, 0.821). At follow-up CCTA, logistic regression analysis showed that increase in fat attenuation index (FAI) and decrease in PCAT volume were independent predictors of PP. The predictive capability of the combined model (increase in FAI + decrease in PCAT volume) was the best in the training and validation sets (AUC = 0.907, 0.882).

**Conclusions:**

At baseline CCTA, the RF-based PCAT radiomics model demonstrated excellent predictive ability for PP. Furthermore, at follow-up CCTA, our results indicated that both increase in FAI and decrease in PCAT volume can independently predict PP, and their combination provided enhanced predictive ability.

## 1 Introduction

Cardiovascular disease remains the leading cause of death worldwide [1]. Atherosclerotic plaque progression (PP) is considered a key step between early-stage or uncomplicated atherosclerosis and eventual plaque rupture [2]. The progression, erosion, and rupture of coronary atherosclerotic plaques are closely linked to major adverse cardiovascular events (MACE) [3]. Some studies have indicated that PP often precedes MACE [4], [5], [6]. However, a few studies have specifically focused on PP and indicated factors potentially linked to it. Therefore, accurate and early identification of PP is crucial, and clinicians should take proactive and effective intervention measures to reduce the incidence of MACE.

Currently, intravascular ultrasound (IVUS) and optical coherence tomography (OCT) are acknowledged as the gold standards for evaluating coronary plaques [7]. However, both methods are invasive and costly. In contrast, coronary computed tomography angiography (CCTA) is the preferred non-invasive tool for assessing coronary plaques and has shown a strong correlation with IVUS in quantitative plaque analysis [8]. In comparison to IVUS analysis, CCTA provides remarkable sensitivity and accuracy in identifying plaque and morphological features [9]. CCTA-derived conventional parameters, including quantitative and qualitative plaque characteristics, computed tomography fractional flow reserve (CT-FFR), epicardial adipose tissue (EAT), and fat attenuation index (FAI), have been employed to predict PP, but their predictive efficacy remains limited [10], [11], [12], [13]. Combining machine learning with radiomics enables artificial intelligence to uncover hidden patterns in large, complex datasets, thereby generating more efficient predictive models than traditional methods. Radiomics offers a new frontier in cardiac CT research, as it allows the extraction of numerous features from images that are invisible to the human eye, enabling quantitative data analysis [14]. Previous studies have demonstrated that pericoronary adipose tissue (PCAT) radiomics significantly improves MACE prediction and offers incremental value in diagnostic performance for identifying flow-limiting coronary stenosis [15], [16]. Moreover, increase in inflammation of PCAT is independently associated with PP [17]. However, to our knowledge, few studies have evaluated the efficacy of different machine learning models of PCAT radiomics in predicting PP.

Therefore, the main purpose of our study was to evaluate different machine learning models of PCAT radiomics and to develop and validate the best model for predicting PP.

## 2 Materials and methods

### 2.1 Study population

The study protocol met international ethical standards and complied with the Declaration of Helsinki. In addition, this retrospective study was approved by the institutional research ethics committee of General Hospital of Central Theater Command of People's Liberation Army (review number: [2024]061–01), and the requirement to obtain informed consent was waived. Measures were implemented to anonymize the data, protect patients’ privacy, and ensure the secure storage of data.

Patients with suspected or known coronary artery disease (CAD) were enrolled who underwent at least two CCTA examinations between January 2016 and June 2022. The exclusion criteria were: (1) interscan interval between baseline and follow-up CCTA examinations was less than one year; (2) patients with percutaneous coronary intervention (PCI) or coronary artery bypass grafting (CABG) before baseline or follow-up CCTA examination; (3) poor image quality; (4) insufficient clinical or imaging data; (5) no plaques at the baseline CCTA examination.

### 2.2 CCTA protocol

CCTA scans were performed using a 320-detector-row CT scanner (Aquilion ONE, Toshiba, Tokyo, Japan). The same equipment was used for both CCTA examinations to ensure consistency in tube voltage. All subjects received 0.5 mg sublingual nitroglycerin five minutes before CCTA acquisition. For patients with a heart rate ≥ 70 bpm, oral metoprolol tablets should be taken to control the heart rate to be 60–70 bpm. A bolus of 60 mL iodinated contrast media (indixanol, 320 mg/mL, Jiangsu Hengrui) was injected at 5 mL/s followed by a 30 mL saline flush. The regions of interest were selected within the ascending aorta. CCTA acquisition was triggered at a 4-second delay after the peak time of the ascending aorta. A retrospective ECG-gated protocol was employed for the scan. The scanning parameters were as follows: tube voltage, 100 kVp; reconstructed slice thickness, 0.5 mm; reconstructed slice interval, 0.5 mm.

### 2.3 CCTA image analysis

Two cardiovascular radiologists, with 3 (J.J.P) and 13 (J.N.Z) years of experience, independently analyzed all CCTA datasets using semi-automated plaque analysis software (Vitrea, Version 6.0 with SUREPlaque; Vital Images and Canon Medical Systems) [18], [19]. The software demonstrated a high level of intraobserver and interobserver repeatability in measuring plaque characteristics [19]. It can automatically extract the coronary artery centerline and identify both the inner and outer contours of the plaque and blood vessels, with manual corrections applied if necessary. Additionally, detailed measurement methods have been described in previous study [19]. Target coronary plaques were coregistered between the CCTA-1 and CCTA-2 evaluations using fiduciary landmarks such as distance from the ostium and branch vessels. Patient information was blinded, except for the location of the target plaques. Each plaque parameter was measured twice, and the average value was taken as the final result. All coronary arteries with diameters > 2 mm were evaluated using a modified 18-segment model [20], with analysis limited to major arteries (segments 1, 2, 3, 6, 7, 8, 11, 13, and 15) [21]. Plaque was defined as any tissue > 1 mm³ that could be distinguished from surrounding tissue. For segments with multiple plaques, the progressive plaque was analyzed; if no progression was noted, the largest plaque was used [22]. The following plaque parameters were assessed: diameter stenosis (DS); plaque length; plaque volume (PV); plaque burden (PB). Diameter stenosis (DS) = (reference diameter − minimal lumen diameter) / reference diameter; plaque volume (PV) = vessel volume − lumen volume; plaque burden (PB) = plaque volume / vessel volume. Total PV was further subclassified using predefined Hounsfield units (HU) cutoff values: necrotic core PV (-30–30 HU), fibro-fatty PV (30–130 HU), fibrous PV (131–350 HU), calcified PV (> 350 HU), noncalcified PV (-30–350 HU) = necrotic core PV + fibro-fatty PV + fibrous PV [17]. Longitudinal changes in plaque parameters, were defined as each parameter at follow-up minus that at baseline. PP was defined as total PV that increased by more than 10 % between the baseline and follow-up CCTA [23]. The progressive and nonprogressive plaques were randomly divided into training and validation sets at a ratio of 7:3.

### 2.4 PCAT conventional parameters and radiomics analysis

Based on the method by Oikonomou et al. [24], PCAT segmentation and radiomics feature extraction were conducted using dedicated PCAT analysis software (CoronaryDoc, FAI Analysis Tool, V7.05, ShuKun Technology, Beijing, China) [16], [25], [26]. The software is based on a pre-trained deep learning algorithm, which has been described previously [27]. Within the manually defined segment of interest, the software can automatically calculate PCAT parameters and extract radiomics features, thereby ensuring high repeatability and reliability. PCAT was defined as the adipose tissue extending radially outward from the outer vessel wall, a distance equal to the coronary artery's diameter, with all voxels in the −190 to −30 HU range [24]. FAI was quantified as the mean CT attenuation of the adipose tissue. All PCAT segmentation was performed by trained operators (J.J.P and J.N.Z, with 3 and 4 years of experience). We measured FAI and PCAT volume in the target peri-plaque region, and the proximal 40 mm segments of three major epicardial coronary vessels where the target plaque was located (right coronary artery: 10–50 mm, left anterior descending artery and left circumflex artery: 0–40 mm). Four PCAT parameters were derived: peri-plaque FAI, peri-plaque PCAT volume, proximal FAI, and proximal PCAT volume. Longitudinal changes in PCAT conventional parameters, were defined as each parameter at follow-up minus that at baseline.

**Fig. 1:**
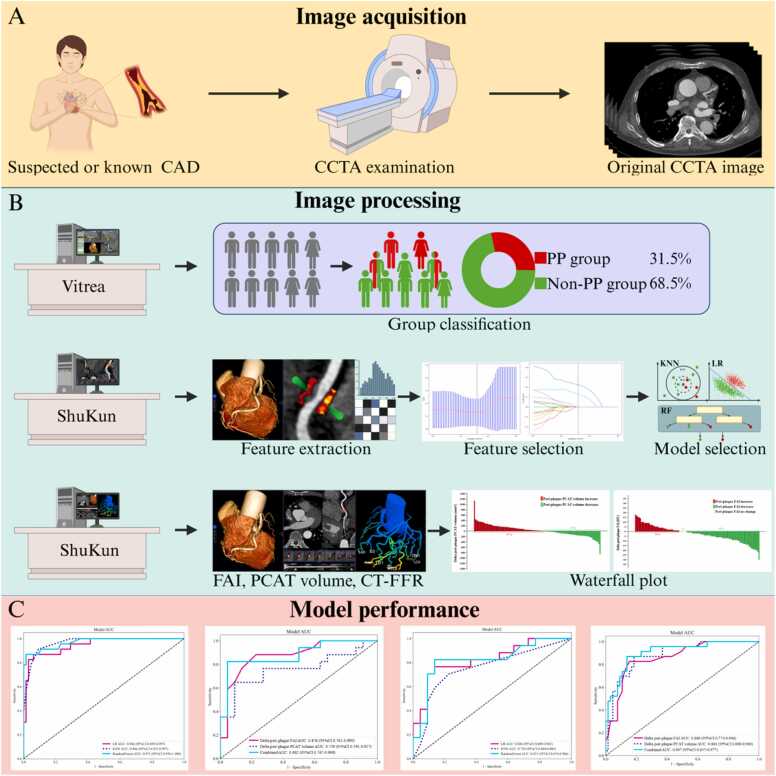
Workflow of this study. CAD, coronary artery disease; CCTA, coronary computed tomography angiography; Vitrea and ShuKun, two kinds of software; PP, plaque progression; KNN, K-nearest neighbors; LR, logistic regression; RF, random forest; FAI, fat attenuation index; PCAT, pericoronary adipose tissue; CT-FFR, computed tomography fractional flow reserve.

Peri-plaque PCAT masks were transferred to an in-house developed radiomics analysis program to extract radiomics features. The workflow of radiomics analysis is shown in Fig. 1. Finally, we extracted 93 radiomics features from each peri-plaque PCAT, including 18 first-order features, 24 gray-level co-occurrence matrix (GLCM) features, 16 gray-level size zone matrix (GLSZM) features, 16 gray-level run length matrix (GLRLM) features, 5 neighboring gray-tone difference matrix (NGTDM) features, and 14 gray-level dependency matrix (GLDM) features. Features with high repeatability were evaluated using Spearman's rank correlation coefficient, which calculated the correlation between the features. If the correlation coefficient between any two features greater than 0.7, one of the features was retained. Additionally, the least absolute shrinkage and selection operator (LASSO) regression was used to further identify the most significant radiomics features, with the optimal lambda determined through tenfold cross-validation.

### 2.5 CT-FFR calculation

CT-FFR calculations were performed by trained operators (J.J.P and J.N.Z, with 3 and 4 years of experience) using dedicated software (CoronaryDoc, CT-FFR Analysis Tool, V7.05, ShuKun Technology, Beijing, China). The Principles and details of CT-FFR have been described in previous study [28]. CT-FFR values specific to ischemia were measured 2 cm distal to the end of the lesion plaque. Longitudinal change in CT-FFR, was defined as CT-FFR at follow-up minus that at baseline.

### 2.6 Radiomics and conventional parameter models’ construction and validation

At baseline CCTA, after dimensionality reduction and feature selection, PCAT radiomics models were constructed by logistic regression (LR), K-nearest neighbors (KNN), and random forest (RF). Machine learning model hyperparameters are listed in Supplementary Table S1. Logistic regression analysis was applied to identify variables for developing conventional parameter models. All model performances were assessed using some metrics such as the area under the curve (AUC), accuracy, sensitivity, and specificity.

### 2.7 Statistical analysis

Statistical analyses and image plotting were performed using IBM SPSS Statistics (version 27.0) and Python (version 3.7.1). Continuous variables were expressed as mean ± SD or median with interquartile range (IQR) as appropriate. Unordered categorical variables were reported as frequencies and percentages. Continuous variables were compared using Student’s *t*-test or the Mann-Whitney *U* test, depending on whether they followed a normal or non-normal distribution. Unordered categorical variables were compared using Fisher's exact test or the chi-square (χ²) test, as appropriate. Independent predictors of PP were identified through univariate and multivariate logistic regression analyses. Relative risks were expressed as odds ratios (OR) with 95 % confidence intervals (CI). These independent predictors were then incorporated into models establishment. Receiver operating characteristic (ROC) curves were generated for each model. A two-tail *P* < 0.05 was considered statistically significant.

## 3 Results

### 3.1 Baseline characteristics

**Fig. 2:**
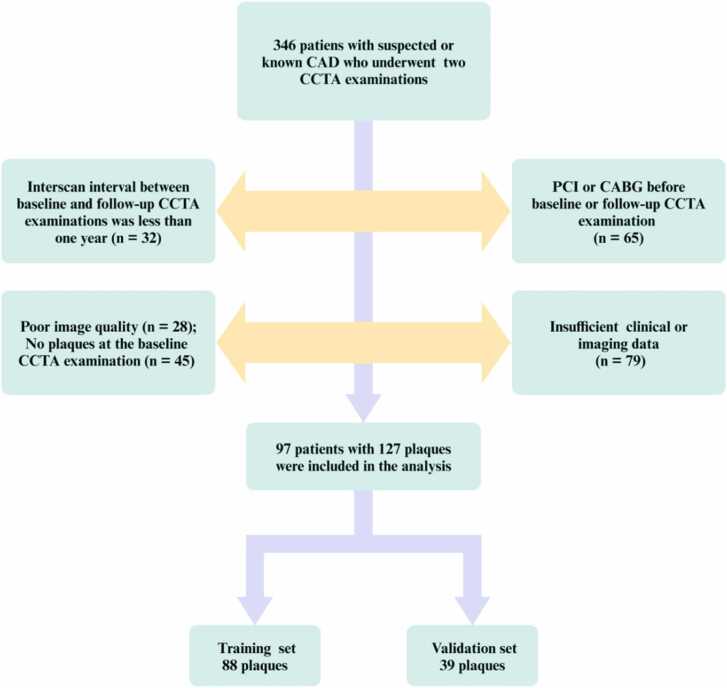
Flowchart of inclusion and exclusion. CAD, coronary artery disease; CCTA, coronary computed tomography angiography; PCI, percutaneous coronary intervention; CABG, coronary artery bypass grafting.

A total of 346 patients with serial CCTA examinations were retrospectively included in the study. However, 249 patients were excluded for the following reasons (Fig. 2): interscan interval between baseline and follow-up CCTA examinations was less than one year (n = 32); PCI or CABG before baseline or follow-up CCTA examination (n = 65); poor image quality (n = 28); insufficient clinical or imaging data (n = 79); no plaques at the baseline CCTA examination (n = 45). Ultimately, 97 patients (mean age 63.9 ± 11.2 years, 68 % males) with 127 plaques were included in the analysis (Table 1). BMI was 24.5 (22.4, 26.7) kg/m² of the study population, and the prevalence of comorbidities included hypertension in 64 % of patients, diabetes mellitus in 28.9 %, hyperlipidemia in 38.1 %, and current smoking in 29.9 %. The interscan interval between the two CCTA examinations was 889 (720, 1474) days.

**Table 1:** Baseline patient characteristics.

Variables	
Age (y)	63.9 ± 11.2
Men, n(%)	66 (68.0)
BMI (kg/m²)	24.5 (22.4, 26.7)
Hypertension, n(%)	62 (64.0)
Diabetes mellitus, n(%)	28 (28.9)
Hyperlipidemia, n(%)	37 (38.1)
Current smoking, n(%)	29 (29.9)
Total cholesterol (mmol/L)	4.1 (3.6, 5.0)
Low-density lipoprotein (mmol/L)	2.2 (1.7, 2.5)
High-density lipoprotein (mmol/L)	1.1 (1.0, 1.4)
Triglycerides (mmol/L)	1.4 (0.9, 2.0)
Lipoprotein (a) (mg/L)	126.0 (78.5, 232.5)
CCTA examination interval (d)	889 (720, 1474)

Data are mean ± standard deviation, n (%), or median (interquartile range).

BMI, body mass index; CCTA, coronary computed tomography angiography.

Of the 127 plaques, 40 (31.5 %) exhibited PP at follow-up CCTA. The progressive and nonprogressive plaques were randomly divided into training and validation sets at a ratio of 7:3, with 88 plaques in the training set and 39 in the validation set (Table S2). The baseline lesion characteristics and characteristics change on CCTA between the PP and non-PP groups are presented in Table 2. At baseline CCTA, compared with non-PP group, PP group exhibited higher peri-plaque PCAT volume (1696.5 vs. 719.0 mm³, *P* < 0.001), necrotic core PV (36.8 vs. 28.2 mm³, *P* = 0.047), fibro-fatty PV (128.0 vs. 83.3 mm³, *P* = 0.026), noncalcified PV (248.3 vs. 167.4 mm³, *P* = 0.026), total PV (263.5 vs. 176.0 mm³, *P* = 0.015), and longer plaque length (29.4 vs. 16.4 mm, *P* < 0.001). However, some unexpected results were observed: the PP group had significantly lower proximal FAI (-79.3 vs. −71.9 HU, *P* < 0.001), peri-plaque FAI (-84.0 vs. −71.0 HU, *P* < 0.001), and plaque burden (53.1 vs. 58.3, *P* = 0.005) compared to the non-PP group. Furthermore, as expected, the PP group also had a lower CT-FFR (0.92 vs. 0.94, *P* = 0.013) than those in the non-PP group. No significant differences were observed between the two groups in terms of proximal PCAT volume, DS, fibrous PV, or calcified PV (all *P* > 0.05). At follow-up CCTA, PP group exhibited higher increase in proximal FAI (3.0 vs. −6.0 HU, *P* < 0.001) and peri-plaque FAI (4.9 vs. −6.0 HU, *P* < 0.001), along with significant decrease in proximal PCAT volume (-94.7 vs. 168.3 mm³, *P* < 0.001) and peri-plaque PCAT volume (-133.0 vs. 80.0 mm³, *P* < 0.001). Similar trends were observed in the changes of plaque characteristics between the two groups. There was no significant difference in the change of CT-FFR between the two groups (*P* = 0.246).

**Table 2:** Baseline lesion characteristics and characteristics change.

Variables	PP group (n = 40)	Non-PP group (n = 87)	*P*
Proximal FAI (HU)	−79.3 ± 10.1	−71.9 ± 8.8	< 0.001
Peri-plaque FAI (HU)	−84.0 (−90.8, −74.0)	−71.0 (−77.0, −63.0)	< 0.001
Proximal PCAT volume (mm³)	1928.1 ± 490.8	1730.2 ± 584.1	0.065
Peri-plaque PCAT volume (mm³)	1696.5 (712.0, 2008.3)	719.0 (404.0, 1071.0)	< 0.001
CT-FFR	0.92 (0.86, 0.96)	0.94 (0.92, 0.97)	0.013
DS (%)	41.0 (30.0, 57.0)	35.0 (28.0, 48.0)	0.114
Plaue length (mm)	29.4 (20.8, 41.2)	16.4 (10.9, 24.9)	< 0.001
Necrotic core PV (mm³)	36.8 (25.0, 62.5)	28.2 (16.4, 48.3)	0.047
Fibro-fatty PV (mm³)	128.0 (77.7, 201.9)	83.3 (55.9, 152.8)	0.026
Fibrous PV (mm³)	66.7 (32.6, 118.7)	41.8 (25.5, 81.3)	0.065
Noncalcified PV (mm³)	248.3 (126.8, 369.9)	167.4 (105.0, 275.0)	0.026
Calcified PV (mm³)	7.3 (0, 45.5)	3.9 (0.0, 15.0)	0.114
Total PV (mm³)	263.5 (157.8, 426.3)	176.0 (106.0, 281.0)	0.015
Plaque burden (%)	53.1 ± 8.1	58.3 ± 10.2	0.005
Delta proximal FAI (HU)	3.0 (−1.8, 7.5)	−6.0 (−9.0, −2.0)	< 0.001
Delta peri-plaque FAI (HU)	4.9 ± 6.3	−6.0 ± 7.6	< 0.001
Delta proximal PCAT volume (mm³)	−94.7 ± 232.2	168.3 ± 278.1	< 0.001
Delta peri-plaque PCAT volume (mm³)	−133.0 (−210.0, −40.0)	80.0 (1.0, 178.0)	< 0.001
Delta CT-FFR	−0.01 (−0.04, 0.04)	−0.01 (−0.03, 0.00)	0.246
Delta necrotic core PV (mm³)	4.5 (−0.5, 11.5)	−2.0 (−6.7, 3.0)	< 0.001
Delta fibro-fatty PV (mm³)	7.3 (−5.3, 25.8)	−12.1 (−27.4, −0.2)	< 0.001
Delta fibrous PV (mm³)	24.9 (0.5, 33.3)	−2.9 (−22.0, 8.3)	< 0.001
Delta non-Calcified PV (mm³)	32.0 (13.4, 50.6)	−14.3 (−52.0, 0.5)	< 0.001
Delta calcified PV (mm³)	12.7 (3.0, 30.3)	2.3 (0.0, 8.9)	< 0.001

Data are mean ± standard deviation, n (%), or median (interquartile range). Variables following a normal distribution are compared using the Student's *t*-test, while those with a non-normal distribution are compared using the Mann-Whitney *U* test. Unordered categorical variables are compared using either Fisher’s exact test or the chi-square (χ²) test. FAI, fat attenuation index; PCAT, pericoronary adipose tissue; CT-FFR, computed tomography fractional flow reserve; DS, diameter stenosis; PV, plaque volume.

### 3.2 Radiomics models for predicting PP, model performance and comparison

**Fig. 3:**
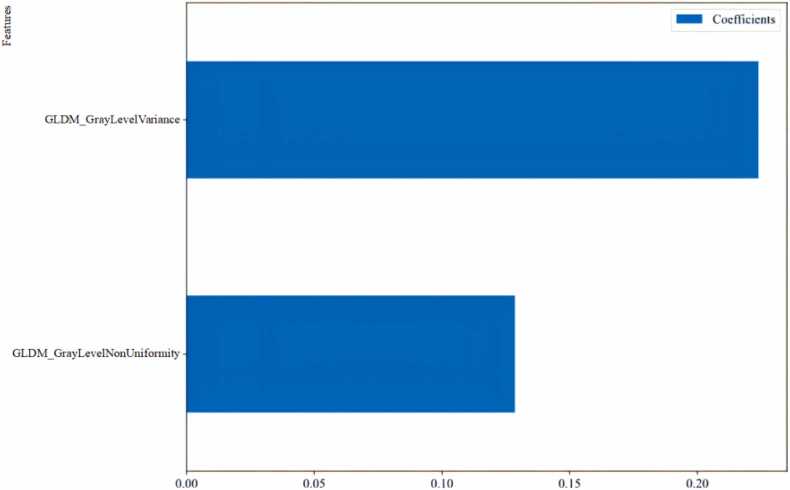
Ranking of peri-plaque PCAT radiomics feature importance. GLDM, gray-level dependence matrix.

**Fig. 4:**
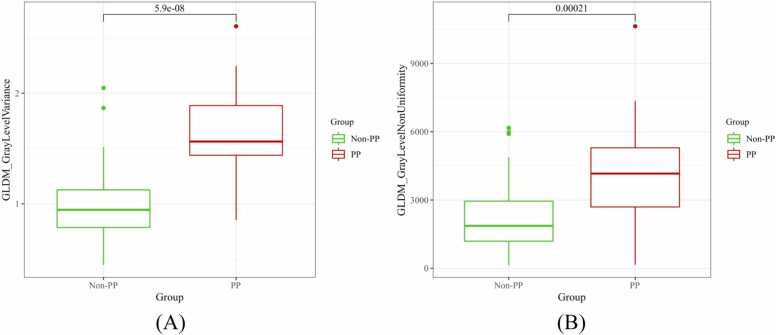
Box plots of the selected radiomics features including GLDM_GrayLevelVariance (A) and GLDM_GrayLevelNonUniformity (B) for PP and non-PP in the training set.

**Fig. 5:**
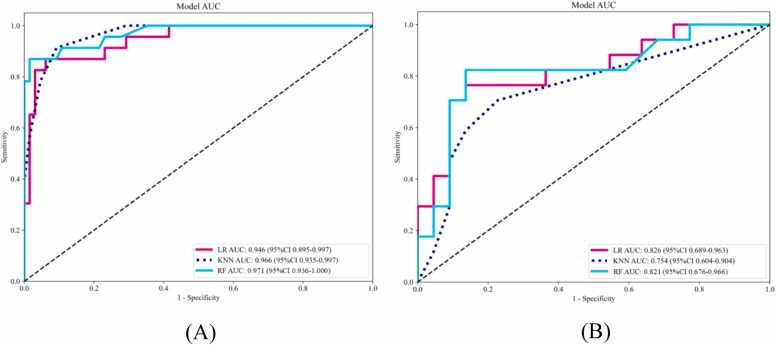
Comparison of receiver operating characteristic curves for the LR, KNN, and RF in the training (A) and validation (B) sets, respectively. LR, logistic regression; KNN, K-nearest neighbors; RF, random forest.

A total of 93 radiomics features were extracted from CCTA images. Two radiomics features, GLDM_GrayLevelVariance and GLDM_GrayLevelNonUniformity, were selected as the most valuable features using LASSO regression, with their individual coefficients presented in Fig. 3. Fig. 4 demonstrates the box plots of the selected radiomics features for PP and non-PP in the training set. Subsequently, these features were input into three machine learning models for analysis. The ROC curves for the three radiomics models in both the training and validation sets are presented in Fig. 5. The AUC, accuracy, sensitivity, and specificity for each model are detailed in Table 3. At baseline CCTA, the radiomics models showed strong performance in the training set, the AUCs for LR, KNN, and RF were 0.946, 0.966, and 0.971, respectively, with corresponding accuracies of 0.909, 0.909, and 0.943; in the validation set, the AUCs were 0.826, 0.754, and 0.821, with accuracies of 0.795, 0.744, and 0.821. Notably, the KNN model exhibited a trend of overfitting between the training and validation sets. After comprehensive analysis of predictive metrics, the RF model was selected as the optimal radiomics model due to its superior stability, accuracy, and sustainability.

**Table 3:** Diagnostic performance of different models for predicting plaque progression in training and validation sets.

Model		Accuracy	AUC (95 % CI)	Sensitivity	Specificity
LR	Training	0.909	0.946 (0.895, 0.997)	0.826	0.938
	Validation	0.795	0.826 (0.689, 0.963)	0.706	0.864
KNN	Training	0.909	0.966 (0.935, 0.997)	0.783	0.954
	Validation	0.744	0.754 (0.604, 0.904)	0.588	0.864
RF*	Training	0.943	0.971 (0.936, 1.000)	0.826	0.985
	Validation	0.821	0.821 (0.676, 0.966)	0.765	0.864

* Represents the best model. AUC, area under the curve; LR, logistic regression; KNN, K-nearest neighbors; RF, random forest.

### 3.3 Association of conventional parameters and PP

**Fig. 6:**
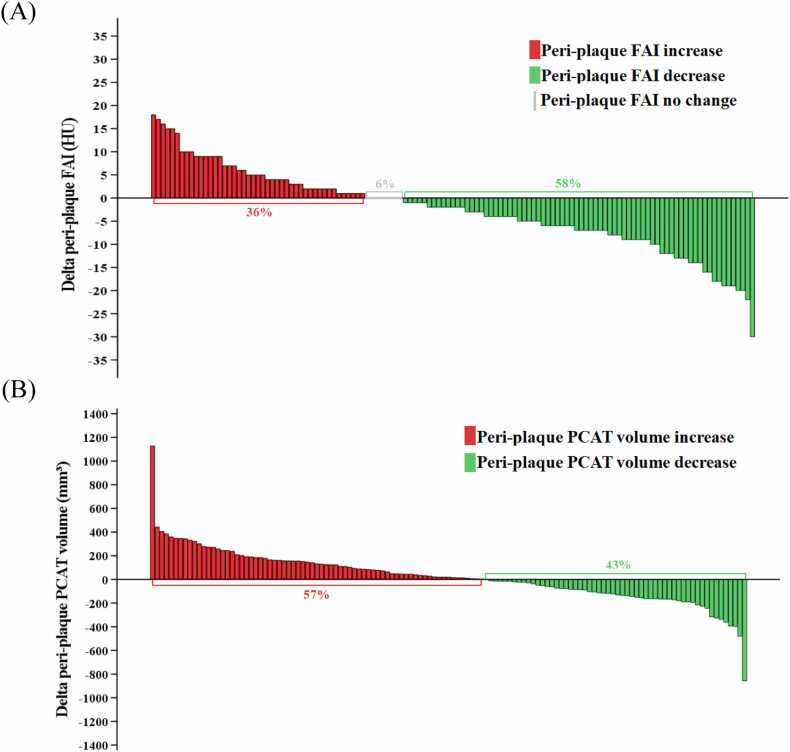
Waterfall plots show the change in (A) peri-plaque FAI and (B) peri-plaque PCAT volume between baseline and follow-up CCTA examinations in the total set. FAI, fat attenuation index; PCAT, pericoronary adipose tissue.

At follow-up CCTA, the univariate logistic regression analysis of delta conventional parameters (follow-up CCTA minus baseline CCTA) revealed that delta proximal FAI, delta peri-plaque FAI, delta proximal PCAT volume, and delta peri-plaque PCAT volume were significantly associated with PP (all *P* < 0.001). When included in the multivariate logistic regression analysis, delta peri-plaque FAI (OR: 1.208, 95 % CI: 1.015–1.438, *P* = 0.033) and delta peri-plaque PCAT volume (OR: 0.992, 95 % CI: 0.986–0.999, *P* = 0.017) were identified as independent predictors of PP (Table 4). Approximately 36 % of peri-plaque FAI showed increase, while 43 % of peri-plaque PCAT volume exhibited decrease (Fig. 6).

**Table 4:** Univariate and multivariate logistic regression analyses of CCTA-derived parameters for predicting plaque progression in the training set.

Varibles	Univariate analysis		Multivariate analysis	
	OR (95 % CI)	*P*	OR (95 % CI)	*P*
Delta proximal FAI (HU)	1.188 (1.086, 1.300)	< 0.001	0.974 (0.834, 1.138)	0.743
Delta peri-plaque FAI (HU)	1.270 (1.134, 1.422)	< 0.001	1.208 (1.015, 1.438)	0.033
Delta proximal PCAT volume (mm³)	0.995 (0.993, 0.998)	< 0.001	0.999 (0.996, 1.002)	0.504
Delta peri-plaque PCAT volume (mm³)	0.988 (0.983, 0.994)	< 0.001	0.992 (0.986, 0.999)	0.017

OR, odds ratio; FAI, fat attenuation index; PCAT, pericoronary adipose tissue.

### 3.4 Conventional delta parameter models for predicting PP, model performance and comparison

**Fig. 7:**
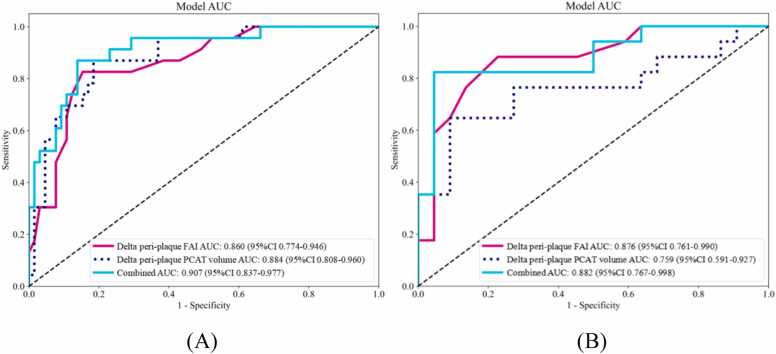
Comparison of receiver operating characteristic curves for the delta peri-plaque FAI model (increase in FAI), delta peri-plaque PCAT volume model (decrease in PCAT volume), and combined model (increase in FAI + decrease in PCAT volume) in the training (A) and validation (B) sets. FAI, fat attenuation index; PCAT, pericoronary adipose tissue.

At follow-up CCTA, three delta models were constructed using logistic regression. The ROC curves for the three delta models in both the training and validation sets are presented in Fig. 7. The AUC, accuracy, sensitivity, and specificity for each model are detailed in Table 5. In the training set, the AUCs for the delta peri-plaque FAI model (increase in FAI), delta peri-plaque PCAT volume model (decrease in PCAT volume), and combined model (increase in FAI + decrease in PCAT volume) were 0.860, 0.884, and 0.907, respectively, with corresponding accuracies of 0.841, 0.818, and 0.852; in the validation set, the AUCs were 0.876, 0.759, and 0.882, with accuracies of 0.821, 0.769, and 0.872, respectively. Compared to the delta peri-plaque PCAT volume model, the delta peri-plaque FAI model displayed greater stability. A comprehensive analysis of the predictive metrics indicated that the combined model offered the best predictive capability.

**Table 5:** Diagnostic performance of different models for predicting plaque progression in training and validation sets.

Variables		Accuracy	AUC (95 % CI)	Sensitivity	Specificity
Delta peri-plaque FAI	Training	0.841	0.860 (0.774, 0.946)	0.739	0.877
	Validation	0.821	0.876 (0.761, 0.990)	0.824	0.818
Delta peri-plaque PCAT volume	Training	0.818	0.884 (0.808, 0.960)	0.826	0.815
	Validation	0.769	0.759 (0.591, 0.927)	0.588	0.909
Combined	Training	0.852	0.907 (0.837, 0.977)	0.826	0.862
	Validation	0.872	0.882 (0.767, 0.998)	0.765	0.955

FAI, fat attenuation index; PCAT, pericoronary adipose tissue.

## 4 Discussion

This study explored the relationship between PCAT radiomics, changes in conventional parameters, and PP. The main finding demonstrated that three different machine learning models of PCAT radiomics were valuable in predicting PP, with the RF model showing superior predictive performance (AUC, training set: 0.971; validation set: 0.821). Additionally, our results indicated that increase in FAI and decrease in PCAT volume were independent predictors of PP, and their combination provided enhanced predictive ability (AUC, training set: 0.907; validation set: 0.882). These findings have important implications for early identification and risk assessment of progressive plaques on CCTA.

Compared to clinical visual assessments, radiomics offers greater utility by providing more objective and detailed information about lesion heterogeneity. This allows for the detection of previously unidentified disease progression and pathophysiological changes within the lesion, particularly when analyzing large datasets [14], [29]. Therefore, radiomics enhances our understanding of the natural history of CAD and PP. PCAT promotes the progression of atherosclerosis by secreting adipocytokines from inflamed adipocytes, and the FAI serves as a quantitative marker for inflammation. However, the accuracy of FAI can be influenced by tube voltage settings [30], which may potentially impact its ability to predict PP. Inflammation causes morphological change in PCAT, such as increased water retention in adipocytes, which can be detected through radiomics analysis. FAI does not account for the complex spatial relationships between voxels, while PCAT radiomics can provide the spatial distribution of voxel gray-level intensities and a quantification of heterogeneity. Consequently, PCAT radiomics may offer valuable and precise information for predicting PP. In our study, GLDM_GrayLevelVariance and GLDM_GrayLevelNonUniformity emerged as the key radiomics features correlating with PP. GLDM_GrayLevelVariance measures the variance of gray levels, indicating the range of gray-level distribution. It captures the degree of the adipose tissue variation, which reveals its complexity or heterogeneity. During PP, PCAT may become more complex or heterogeneous, with pathological changes manifesting as increased gray-level variance in imaging. This increased tissue complexity correlates with the PP, linking GLDM_GrayLevelVariance to PP. GLDM_GrayLevelNonUniformity quantifies the non-uniformity of gray levels, reflecting the distribution non-uniformity of adipose tissue. As the PCAT distribution becomes more uneven, which leads to increased gray-level non-uniformity, the plaques may form and progress gradually. This suggested that increased PCAT texture complexity on imaging, reflected in GLDM_GrayLevelNonUniformity, is associated with PP.

RF, composed of multiple decision trees, is highly capable of managing complex nonlinear relationships [31]. Since PCAT data may exhibit nonlinear characteristics, LR, a linear model, struggles with such complexity, whereas KNN can handle nonlinear relationships to some extent. In terms of noise robustness, both LR and KNN are susceptible to noisy data, potentially reducing their performance [32]. In contrast, RF effectively mitigates the impact of noisy data by aggregating outputs from multiple decision trees, preventing noise in one tree from affecting the entire model. Regarding accuracy and predictive performance, RF uses bootstrap aggregation and randomization of predictors to achieve a high degree of predictive accuracy [33]. RF aggregates predictions from multiple decision trees, even if one decision tree makes an incorrect prediction, the other decision trees can correct it. RF also has strong anti-overfitting capabilities [31], as each decision tree is independently trained on different subsets of the data. In contrast, KNN lacks mechanisms to prevent overfitting, possibly because it is a nonparametric classifier that relies on proximity methods to differentiate between classes [34]. Furthermore, a small K value (e.g., K = 3, as selected in our study) tends to exacerbate overfitting due to the model’s heightened sensitivity to noise in the training data [35]. Although we consider other values of K, we get relatively good results when K = 3. Finally, the relatively small sample size of our study, combined with KNN’s sensitivity to data imbalances, may further limit the model's generalization ability and exacerbate overfitting.

In the previous analysis of the PARADIGM registry primarily involving non-obstructive CAD patients, Lee et al. [17] reported that the increase in perivascular adipose tissue density was significantly positively associated with the PP, which provides valuable evidence to support the relationship between increased FAI and coronary atheroma progression. The change of FAI plays a crucial role throughout the natural history of CAD, as inflammatory changes in the vessel wall often occur in the early stages of atherosclerosis, preceding morphological alterations in the plaque itself [36]. Therefore, clinicians should consider increase in FAI as a significant indicator of coronary inflammation and plaque instability, which enables the early identification of high-risk patients for timely intervention. In a retrospective study by Feng et al. [11], baseline FAI values (− 70.22 ± 7.40 vs. − 73.23 ± 8.50, *P* = 0.001) were higher in the PP group compared to the non-PP group. After multivariate adjustment, FAI (OR: 1.049, 95 % CI: 1.017–1.082, *P* = 0.002) remained an independent predictor of PP. However, our study did not obtain similar results. This discrepancy may be attributed to various factors influencing FAI, such as a history of clinical treatment. Dai et al. [37] explored the effects of statin therapy on serial change of FAI and concluded that patients who initiated statin therapy after baseline CCTA showed decrease in FAI at follow-up CCTA. However, their study excluded patients who had already been on statin therapy prior to the baseline CCTA. In contrast, our study included patients who may have received statin therapy before baseline CCTA. Moreover, compared to the non-PP group, a larger proportion of patients in the PP group may have received statin therapy before baseline CCTA. As a result, the mean FAI value at baseline CCTA was lower in the PP group. During the follow-up period, even after accounting for the effects of statin therapy, patients predisposed to PP demonstrated a gradual increase in coronary artery inflammation, as reflected by increased FAI value at follow-up CCTA. Similar findings were observed in the previous study by Lee et al. [17].

To our knowledge, few studies have reported on the relationship between change in PCAT volume and PP in a longitudinal CCTA study. Our findings indicated that decrease in PCAT volume was an independent predictor of PP, while increase in PCAT volume was indicative of plaque regression or stabilization. These findings are consistent with existing theories. PP is often driven by increased inflammation, and an inflamed coronary artery wall may release inflammatory mediators that exert paracrine effects on PCAT, inhibiting the differentiation of preadipocytes into mature adipocytes, which are larger in volume [38], [39], [40], [41]. Another explanation is that increased inflammation in the PP group causes PCAT to shift toward the aqueous phase, which leads to lower mean PCAT volume compared to the non-PP group. Psaltis et al. [12] argued that epicardial fat volume (EFV) promotes atherosclerotic plaque development, with higher baseline EFV associated with plaque volume progression. However, their study did not measure EFV at follow-up CCTA. Our research made up for this deficiency by performing continuous PCAT volume measurements and demonstrated that reduction in PCAT volume, rather than accumulation, was associated with PP. Although local PCAT volume may not fully represent the entire EFV, changes in PCAT and plaque are likely more strongly correlated due to its closer proximity to the vessel wall [42], providing valuable predictive insights. Moreover, as peri-plaque PCAT is located closer to the plaque than the proximal 40 mm segments PCAT of coronary vessel, our study found that the delta peri-plaque FAI and delta peri-plaque PCAT volume were independent predictors of PP.

Available evidence demonstrated that PP was associated with an increased incidence of MACE. In the previous study reported by Yang et al. [5], a significantly higher proportion of patients in the MACE group exhibited PP compared to those in the no-MACE group [40 (89 %) vs. 89 (37 %), *P* < 0.001], PP was associated with a ninefold increase in the risk of MACE [HR (95 % CI): 9 (3.5–23), *P* < 0.001]. Gu et al. [43] explored the association between PP and cardiovascular events among patients diagnosed with nonobstructive coronary artery disease, and they concluded that three vessel PP [HR (95 % CI): 2.37 (1.39–420), *P* = 0.026] and severe proximal PP [HR (95 % CI): 3.65 (1.56–8.13), *P* = 0.003] were significant predictors of MACE. However, our study aimed to explore the role of PCAT in identifying patients with a high risk of PP, thereby facilitating early clinical interventions and treatments. Further studies are essential to evaluate whether baseline PCAT radiomics or delta PCAT radiomics can reliably predict subsequent MACE. A deeper understanding of the predictive value of these radiomic features could advance future clinical decision-making processes and ultimately improve patient outcomes.

Although our study presented novel findings, several limitations should be acknowledged. First, selection bias was inevitable. Since our study was retrospective, we excluded patients with missing clinical or imaging data and high-risk individuals who had undergone revascularization (PCI or CABG). Additionally, most of our patients had only mild stenosis. Therefore, the findings may primarily reflect outcomes in low-risk populations. Second, PP is a long-term process; however, the follow-up period in our study is relatively short (minimum 1 year). Future studies could obtain more precise and detailed information about the amount and rate of PP by using a longer follow-up period. Third, the time intervals between the CCTA-1 and CCTA-2 examinations exhibit a relatively wide range, potentially introducing time-dependent bias. Therefore, future studies should consider conducting subgroup analyses based on different follow-up times. Fourth, the radiomic features (GLDM_GrayLevelVariance and GLDM_GrayLevelNonUniformity) selected in our study lack sufficient reference in early cardiovascular imaging; therefore, further studies are needed to explore the biological significance of these radiomic features. Fifth, external validation was not performed, which should be conducted in multiple centers to enhance the robustness of our findings. Sixth, clinical outcomes after the second CCTA were not collected, limiting our ability to assess the association between PCAT radiomics, change in FAI and PCAT volume, and patient clinical outcomes. Seventh, our plaque analyses were limited to the three major coronary arteries and did not include branch vessels. Finally, due to challenges in collecting comprehensive medication data, the patients' medication use was not documented.

## 5 Conclusions

In conclusion, at baseline CCTA, the PCAT radiomics model using RF exhibited excellent predictive ability for PP. Additionally, at follow-up CCTA, the increase in FAI and decrease in PCAT volume were significant indicators of PP, and their combination provided enhanced predictive ability. These findings suggest a potential non-invasive method for early identification and risk assessment of PP.

## Funding statement

This study was supported by the Yuying Plan Project of General Hospital of Central Theater Command of People's Liberation Army (grant number: ZZYFH202101).

## Ethical statement

The study protocol met international ethical standards and complied with the Declaration of Helsinki. This study was approved by the institutional research ethics committee of General Hospital of Central Theater Command of People's Liberation Army (review number: [2024]061–01). Since this study was retrospective, the requirement for obtaining informed consent was waived. Measures were implemented to anonymize the data, protect patients’ privacy, and ensure the secure storage of data.

## CRediT authorship contribution statement

**Zou Jiani:** Writing – review & editing, Writing – original draft, Funding acquisition, Data curation, Conceptualization. **Peng Shuhui:** Writing – review & editing. **Huang Wencai:** Writing – review & editing, Conceptualization. **Zhu Jiangming:** Writing – review & editing, Supervision. **Fu Tingting:** Writing – review & editing. **Wu Qian:** Writing – review & editing, Supervision. **Huang Qianyu:** Writing – review & editing, Writing – original draft, Visualization. **Pan Jingjing:** Writing – review & editing, Writing – original draft, Visualization, Data curation.

## Declaration of Competing Interest

The authors declare that they have no relevant financial or non-financial interests to disclose.
