# Supplementary material for: Prediction of plaque progression using different machine learning models of pericoronary adipose tissue radiomics based on coronary computed tomography angiography
Source: Eur J Radiol Open. 2025 Feb 15;14:100638. doi: 10.1016/j.ejro.2025.100638 (PMC11872547; doi:10.1016/j.ejro.2025.100638)

**Table S1**

Various hyperparameters of machine learning models for predicting plaque progression.

| Machine Learning Model | Hyperparameters |
| --- | --- |
| LR | Penalty='none'  Max_iter=100 |
| KNN | n_Neighbors=3  Weights=‘distance’  Distance Metric=Minkowski (p=2)  n_Jobs=4 |
| RF | n_Estimators=5  Max_depth=3  Min_samples_split=4  Random_state=0 |

LR, logistic regression; KNN, K-nearest neighbors; RF, random forest.

**Table S2**

Baseline lesion characteristics and characteristics change.

| Variables | Training set (n = 88) | Validation set (n = 39) | *P* |
| --- | --- | --- | --- |
| Proximal FAI (HU) | -74.8 ± 9.7 | -72.8 ± 10.2 | 0.286 |
| Peri-plaque FAI (HU) | -74.6 ± 11.1 | -74.9 ± 12.0 | 0.880 |
| Proximal PCAT volume (mm³) | 1781.8 ± 569.7 | 1816.6 ± 551.0 | 0.749 |
| Peri-plaque PCAT volume (mm³) | 817.0 (450.3, 1260.8) | 1041.0 (560.0, 1591.0) | 0.266 |
| CT-FFR | 0.94 (0.90, 0.96) | 0.94 (0.90, 0.96) | 0.910 |
| DS (%) | 39.0 (29.0, 51.0) | 33.0 (23.0, 42.0) | 0.061 |
| Plauqe length (mm) | 18.9 ( 11.4, 28.5) | 20.3 ( 11.4, 38.0) | 0.299 |
| Necrotic core PV (mm³) | 32.3 ( 18.6, 51.8) | 33.4 ( 15.0, 62.9) | 0.925 |
| Fibro-fatty PV (mm³) | 91.7 (58.6, 159.3) | 127.2 (55.9, 181.3) | 0.204 |
| Fibrous PV (mm³) | 46.2 (26.6, 98.3) | 53.9 (24.6, 83.7) | 0.996 |
| Noncalcified PV (mm³) | 192.1 ( 112.1, 285.4) | 221.7 ( 102.0, 302.3) | 0.412 |
| Calcified PV (mm³) | 5.0 (0.0, 26.3) | 4.0 (0.0, 12.2) | 0.409 |
| Total PV (mm³) | 198.0 ( 122.5, 342.8) | 226.0 ( 103.0, 311.0) | 0.576 |
| Plaque burden (%) | 58.7 ± 10.1 | 52.1 ± 7.5 | <0.001 |
| Delta proximal FAI (HU) | -3.0 (-8.0, 2.75) | -6.0 (-11.0, 4.0) | 0.203 |
| Delta peri-plaque FAI (HU) | -3.0 ± 8.6 | -1.7 ± 9.2 | 0.448 |
| Delta proximal PCAT volume (mm³) | 52.2 ± 258.1 | 160.5 ± 345.5 | 0.085 |
| Delta peri-plaque PCAT volume (mm³) | 14.5 (-86.0, 132.0) | 45.0 (-122.0, 202.0) | 0.507 |
| Delta CT-FFR | -0.01 (-0.03, 0.01) | 0.00 (-0.04, 0.01) | 0.755 |
| Delta necrotic core PV (mm³) | -1.0 (-5.8, 4.9) | 2.0 (-4.1, 6.4) | 0.374 |
| Delta fibro-fatty PV (mm³) | -6.2 (-21.1, 8.1) | -2.7 (-41.8, 7.5) | 0.644 |
| Delta fibrous PV (mm³) | 1.2 (-13.9, 20.9) | 2.8 (-19.8, 16.0) | 0.770 |
| Delta non-Calcified PV (mm³) | -4.1 (-31.0, 11.9) | 1.0 (-52.0, 26.6) | 0.756 |
| Delta calcified PV (mm³) | 4.7 (0.0, 15.0) | 6.3 (0.0, 19.9) | 0.485 |

Data are mean ± standard deviation, n (%), or median (interquartile range). Variables following a normal distribution are compared using the Student's t-test, while those with a non-normal distribution are compared using the Mann-Whitney U test. Unordered categorical variables are compared using either Fisher’s exact test or the chi-square (χ²) test. FAI, fat attenuation index; PCAT, pericoronary adipose tissue; CT-FFR, computed tomography fractional flow reserve; DS, diameter stenosis; PV, plaque volume.

**Confirmation of Publication and Licensing Rights**


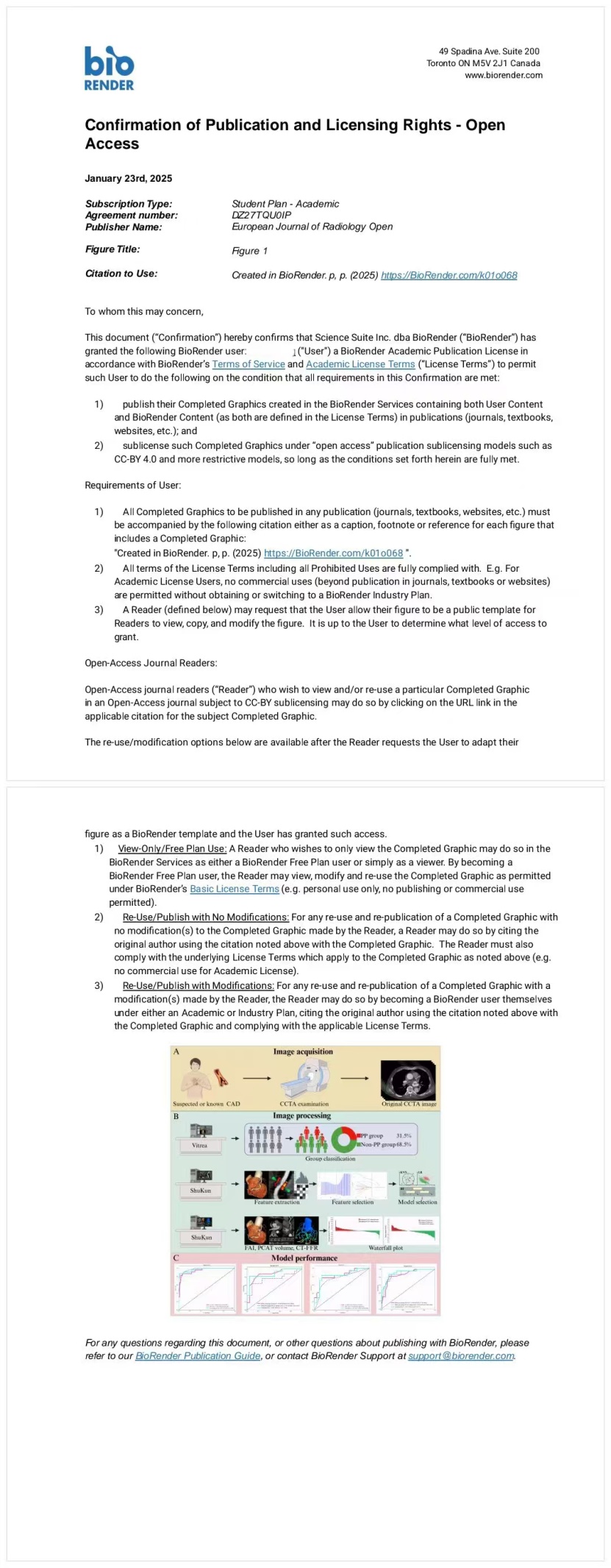


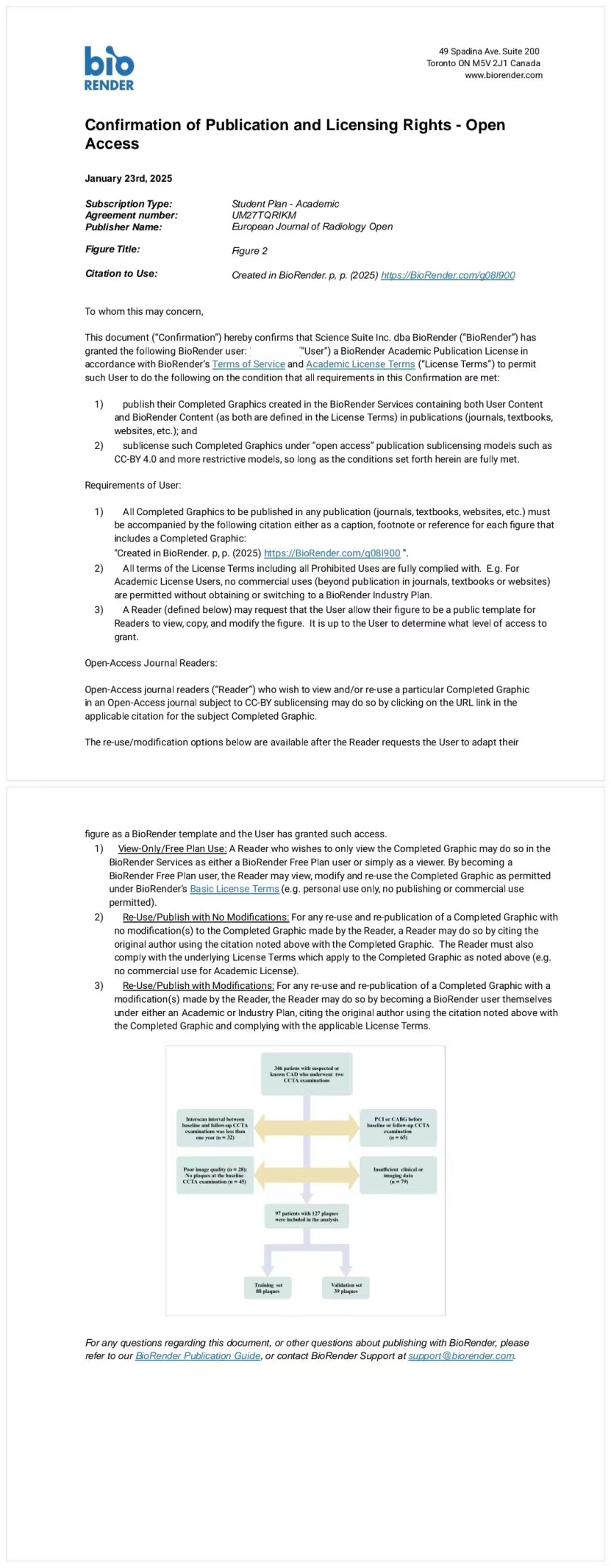

Supplement: Supplementary file 1 — Supplementary material [file mmc1.docx]
